# Venous thromboembolism after ventral hernia repair with transversus abdominis release

**DOI:** 10.1007/s00464-025-12001-y

**Published:** 2025-07-30

**Authors:** Kimberly P. Woo, Sergio Mazzola Poli de Figueiredo, Sarah L. Larson, Joseph D. Quick, Sara M. Maskal, Daphne Remulla, William C. Bennett, Kimberly S. Miles, Chao Tu, Lucas R. Beffa, Clayton C. Petro, Ajita S. Prabhu, David M. Krpata, Michael J. Rosen, Benjamin T. Miller

**Affiliations:** 1 Department of General Surgery, Digestive Disease Institute, Cleveland Clinic, 9500 Euclid Ave, Cleveland, OH 44195 USA; 2 University of Minnesota Medical School, Minneapolis, MN USA; 3 Cleveland Clinic Lerner College of Medicine, Cleveland, OH USA; 4 Department of Quantitative Health Sciences, Lerner Research Institute, Cleveland Clinic, Cleveland, OH USA; 5 Department of General Surgery, Northwestern Medicine, Chicago, IL USA

**Keywords:** Ventral hernia repair, Transversus abdominis release, Abdominal wall reconstruction, Venous thromboembolism, Deep vein thrombosis, Pulmonary embolism

## Abstract

**Introduction:**

Venous thromboembolism (VTE) events, such as pulmonary embolism (PE) and deep venous thrombosis (DVT), are a significant source of morbidity and mortality after major abdominal wall reconstruction. We aim to describe the incidence of VTE events in patients undergoing ventral hernia repair (VHR) with transversus abdominis release (TAR) at our institution.

**Methods:**

The Abdominal Core Health Quality Collaborative registry was queried for patients, 18 years and older, who underwent VHR with TAR at our institution between August 2014 and December 2023. Patient characteristics and operative details were obtained from the registry. Patient electronic medical records were reviewed for outcomes. The primary study outcome was the incidence of VTE, including PE and DVT, within 8 weeks postoperatively. Secondary outcomes included time to VTE, VTE chemoprophylaxis protocol used, VTE management, and complications.

**Results:**

Of the 3555 patients who underwent abdominal wall reconstruction and met inclusion criteria, 100 patients experienced (2.8%) VTE events; 58 patients had a PE and 42 had DVTs. The median time to a VTE event was 7 (IQR 4–14) days after surgery, and 27% of VTE events occurred after hospital discharge. Of the patients who experienced a VTE event, 77 received preoperative chemoprophylaxis and 66 patients received postoperative chemoprophylaxis beginning on the day of surgery. In 19 patients, chemoprophylaxis was interrupted prior to development of VTE. Most patients (*n* = 88) were managed with systemic anticoagulation, and 18.1% had a bleeding complication related to anticoagulation treatment.

**Conclusion:**

Patients undergoing major abdominal wall reconstruction are at high risk for venous thromboembolism events, even after hospital discharge. Further studies are needed to determine risk factors associated with VTE in this specific patient population to optimize perioperative anticoagulation strategies.

Venous thromboembolism (VTE), which includes pulmonary embolism (PE) and deep vein thrombosis (DVT), represents a serious, and potentially fatal, complication after ventral hernia repair (VHR), with an estimated of 0.5–0.92% [1–4]. Although the etiology of VTE after VHR is not fully understood, a number of risk of factors have been identified, including higher body mass index (BMI), prolonged operative time, inpatient surgery, and wider hernia width [3, 5]. While some studies have also identified component separation as a risk factor, others have not found an association [5, 6].

Abdominal wall reconstruction (AWR) with component separation through a posterior transversus abdominis release (TAR) allows for significant myofascial advancement but also includes extensive dissection in the retromuscular space to provide a large pocket for mesh placement [7, 8]. Fascial closure to restore abdominal domain during complex AWR can impose significant physiologic alterations, and patients undergoing AWR have previously been found to be at an increased risk for postoperative complications related to elevated intraabdominal pressures [9–11]. It has been proposed that restoration of the abdominal wall domain after complex AWR may increase intraabdominal pressure, decreasing venous return, and thus increasing venous stasis [12, 13]. Additionally, the prolonged operative times associated with AWR may further predispose patients to an increased risk of VTE [14, 15]. However, the specific incidence of VTE after complex AWR has not been well reported in the literature and ranges widely from 0.5 to 4.1% [15–18]. The purpose of this analysis is to evaluate the incidence of venous thromboembolism after complex abdominal wall reconstruction at a single, high-volume institution and describe the perioperative characteristics associated with postoperative VTE in these patients.

## Methods

This retrospective review was conducted with approval from the Institutional Review Board at the Cleveland Clinic (IRB# 24-539). The Strengthening the Reporting of Observational Studies in Epidemiology (STROBE) reporting guidelines were followed [19].

### Data source and study population

Patients 18 years of age and older, undergoing open or minimally invasive mesh-based ventral hernia repair with transversus abdominis release (TAR) between August 2014 and December 2023 at our institution, were identified using the Abdominal Core Health Quality Collaborative (ACHQC) registry. The ACHQC is a nationwide quality initiative which collects health information related to abdominal core and hernia surgical repairs with the focus of improving patient outcomes [20]. Surgeon-members prospectively enter data including, but not limited to, patient demographics, operative details, short- and long-term follow-up, and patient-reported outcomes.

The patients meeting study inclusion criteria from the ACHQC query were further reviewed to identify study outcomes. VTE events were determined using two sources: a query of 30-day follow-up data in the ACHQC and natural language processing to extract data from the patient electronic medical record using the following key words: “venous thromboembolism,” “pulmonary embolism,” and “deep vein thrombosis.” All patients identified as having a VTE event from these two data sources were confirmed with manual chart review.

### Outcome variables

The primary study outcome was the incidence of VTE within 8 weeks after surgery. A VTE event was defined as an imaging (ultrasound or CT) diagnosis of DVT or PE following TAR. Only the most clinically significant VTE event was counted for patients who were diagnosed with more than one. Patients who were diagnosed with VTE were further reviewed for the following secondary outcomes: type of VTE, time to VTE diagnosis, VTE chemoprophylaxis protocol used, VTE management regimen and complications, intensive care unit (ICU) admission, and mortality.

### Data analysis

Baseline demographics, comorbidities, and operative details from the ACHQC were reviewed. Data were reported as a median (IQR) or mean (SD) for continuous variables and as counts with percentages for categorical variables. The incidence of VTE was calculated as the number of cases of VTE occurring during the study period divided by the population of patients meeting inclusion criteria. The incidence of secondary outcomes was calculated as the number of patients experiencing a given outcome divided by the total number of patients who had a VTE event.

## Results

The query of the ACHQC identified 3555 patients who underwent VHR with TAR at our institution and met inclusion criteria. There was a total of 100 (2.8%) patients who experienced a VTE event. Patient baseline characteristics and operative details are found in Table 1. Those who developed a VTE were older (63.3 ± 10.6 vs 59.4 ± 11.9 years, *p* < 0.001) and more likely to be on anticoagulant medication at baseline (16% vs 7.5%, *p* = 0.003) compared to those who did not. There were a number of significant differences in the operative details between those who developed a VTE and those who did not. For patients who developed a VTE, the operation was less likely be elective (95% vs 99.2%, *p* = 0.002) and more likely to be greater than 240 min (56% vs 32.9%, *p* < 0.001), the hernia was more likely to be recurrent (68% vs 53.8%, *p* = 0.007), and the indication was more likely to be for bowel obstruction (24% vs 11.5%, *p* < 0.001). Additionally, the median hernia length (24.3 vs 22 cm, *p* = 0.002) and width (18.9 vs 15.5 cm, *p* < 0.001) were larger for patients who developed VTE.

**Table 1:** Baseline and operative characteristics of patients undergoing VHR with TAR, with and without VTE

	All *N* = 3555	No VTE *N* = 3455	VTE *N* = 100	*P* value
Baseline characteristics				
Age in years, mean (SD)	59.5 (11.9)	59.4 (11.9)	63.3 (10.6)	<0.001
Gender				0.870
Female	1824 (51.3%)	1774 (51.3%)	50 (50.0%)	
Male	1731 (48.7%)	1681 (48.7%)	50 (50.0%)	
Race				0.167
White	3238 (91.1%)	3147 (91.1%)	91 (91.0%)	
Non-White	317 (8.9%)	308 (8.9%)	9 (9.0%)	
Functional status				0.070
Independent	3501 (98.5%)	3405 (98.6%)	96 (96.0%)	
Partially dependent	46 (1.29%)	42 (1.22%)	4 (4.00%)	
Totally dependent	7 (0.20%)	7 (0.20%)	0 (0.00%)	
BMI, mean (SD)	32.4 (5.95)	32.3 (5.95)	33.4 (5.77)	0.070
Liver failure	25 (0.75%)	24 (0.74%)	1 (1.08%)	0.508
Hypertension	2215 (62.3%)	2143 (62.0%)	72 (72.0%)	0.054
Diabetes	824 (23.2%)	798 (23.1%)	26 (26.0%)	0.577
COPD	346 (9.73%)	334 (9.67%)	12 (12.0%)	0.545
Smoker, current	264 (7.43%)	256 (7.41%)	8 (8.00%)	
Antiplatelet	290 (8.16%)	283 (8.19%)	7 (7.00%)	0.807
Anticoagulant	275 (7.74%)	259 (7.50%)	16 (16.0%)	0.003
Anti-inflammatory	300 (8.44%)	294 (8.51%)	6 (6.00%)	0.479
Operative details				
Elective	3523 (99.1%)	3428 (99.2%)	95 (95.0%)	0.002
Approach				0.431
Laparoscopic	3 (0.08%)	3 (0.09%)	0 (0.00%)	
MIS convert to open	35 (0.98%)	33 (0.96%)	2 (2.00%)	
Open	3398 (95.6%)	3301 (95.5%)	97 (97.0%)	
Robotic	119 (3.35%)	118 (3.42%)	1 (1.00%)	
Wound class				0.109
Clean	2600 (73.1%)	2534 (73.3%)	66 (66.0%)	
Clean-contaminated	505 (14.2%)	489 (14.2%)	16 (16.0%)	
Contaminated	442 (12.4%)	425 (12.3%)	17 (17.0%)	
Dirty/infected	8 (0.23%)	7 (0.20%)	1 (1.00%)	
Operative time in min				<0.001
0–119	367 (10.1%)	361 (10.2%)	6 (6.00%)	
120–179	1045 (29.4%)	1028 (29.8%)	17 (17.0%)	
180–239	948 (26.7%)	927 (26.8%)	21 (21.0%)	
240+	1194 (33.6%)	1138 (32.9%)	56 (56.0%)	
Stoma present	549 (15.4%)	527 (15.3%)	22 (22.0%)	0.089
History of open abdomen	350 (9.85%)	338 (9.78%)	12 (12.0%)	0.573
History of abdominal wall infection	795 (22.4%)	778 (22.5%)	17 (17.0%)	0.236
Recurrent hernia	1925 (54.2%)	1857 (53.8%)	68 (68.0%)	0.007
Hernia length in cm	22.0 (6.44)	22.0 (6.40)	24.3 (7.41)	0.002
Hernia width in cm	15.6 (6.45)	15.5 (6.39)	18.9 (7.69)	<0.001
VHWG grade	2.27 (0.72)	2.27 (0.72)	2.33 (0.73)	0.442
Primary indication for repair				
Symptomatic ventral hernia	3553 (100%)	3453 (100%)	100 (100%)	
Bowel obstruction	421 (11.8%)	397 (11.5%)	24 (24.0%)	<0.001
Fistula	18 (0.51%)	17 (0.49%)	1 (1.00%)	0.402
Infected mesh	28 (0.79%)	27 (0.78%)	1 (1.00%)	0.552
Enlarging or interfering with activities	3075 (86.5%)	2990 (86.6%)	85 (85.0%)	0.761
Pain	3407 (95.9%)	3310 (95.8%)	97 (97.0%)	0.798

### Incidence of VTE

**Fig. 1:**
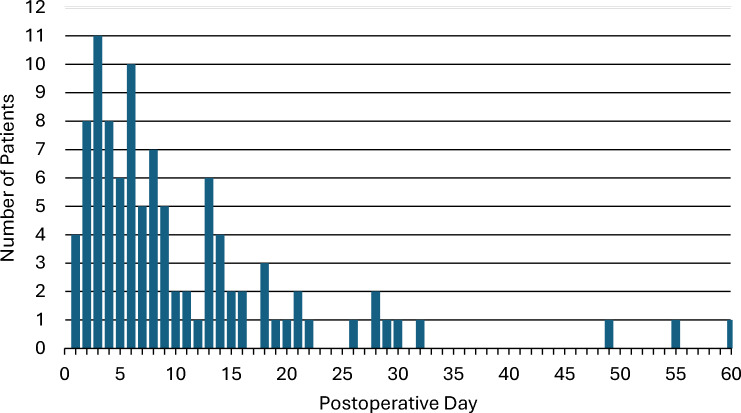
Distribution of patients diagnosed with VTE by postoperative day

For 58 of these patients, the most clinically significant VTE event was a PE, and for 42 patients, it was a DVT. Of the PE events, 11 were subsegmental, 31 segmental, eight lobar, six main, and two saddle. There were 13 patients who developed upper extremity DVTs, 25 who developed lower extremity DVTs, and four who developed internal jugular DVTs. The median time to a VTE event was 7 (IQR 4–14) days after surgery (Fig. 1), and 27% of the VTE events occurred after hospital discharge from index admission.

### VTE chemoprophylaxis

There were 77 patients who received preoperative VTE prophylaxis prior to induction and 23 patients who did not. All patients who developed VTE after surgery received postoperative chemoprophylaxis. Of these patients, 66 were started on postoperative VTE prophylaxis on post-op day (POD) 0 and 34 were started on POD1. One patient received fondaparinux, 16 patients received twice daily subcutaneous heparin, 26 patients received three times daily subcutaneous heparin, 55 patients received once daily enoxaparin, and two patients received twice daily enoxaparin. In 19 patients, chemoprophylaxis was interrupted prior to the development of VTE. Chemoprophylaxis was held for bleeding in 11 patients, a procedure for six patients, and patient refusal for two patients.

### VTE management

The majority of patients with VTE (*n* = 88) were managed with therapeutic anticoagulation. In addition to systemic therapy, nine patients received an inferior vena cava (IVC) filter, three patients underwent endovascular thrombectomy, and one underwent operative thrombectomy. There were eight patients who received an IVC filter only for management of their DVT. Four patients received no additional treatment for their VTE event and were continued only on chemoprophylaxis.

### VTE complications

There were 22 patients who were admitted to the ICU directly related to the VTE event, 10 patients required intubation, and one patient was placed on extracorporeal membrane oxygenation (ECMO). There was one patient who experienced mortality related to their VTE event, which was a main branch PE. Of the patients who received therapeutic anticoagulation, 16 experienced a bleeding complication.

## Discussion

VTE following ventral hernia repair is a serious complication that can confer significant morbidity and mortality. While component separation has been proposed to increase the risk of VTE, there have been limited data on the incidence of VTE in patients undergoing complex AWR [6, 16–18]. Here, we reviewed 3555 patients who underwent VHR with TAR at our institution and identified 100 (2.8%) patients who developed a VTE. Our analysis found that for these patients, the complications after VTE development resulted in a number of complications, including ICU admissions and ventilator requirements, which further increased the morbidity of this high-risk operative procedure.

The incidence of VTE in this study falls within the range of previously reported values after component separation, despite variability in patient data sources and cohort sizes of the prior studies. Kim et al*.* reviewed the American College of Surgeons National Surgical Quality Improvement Program (ACS-NSQIP) database for patients undergoing ventral hernia repair with component separation [6]. Out of 501 patients, there were three (0.5%) patients with a VTE event. This contrasts with the analysis by Nelson et al*.*, also utilizing ACS-NSQIP in a similar patient population which found a rate of 2.1%. Both analyses were conducted using ACS-NSQIP data, for which the accuracy of capturing VTE data has previously been called into question [21]. Kraft and Janis reviewed their own prospectively collected data from patients undergoing complex AWR by a single surgeon at their institution [16]. Four out of their 175 (2.3%) patients were found to have VTE. More recently, Zhang et al*.* reviewed a similar, but updated cohort of 344 patients from the previously described study and found a rate of 4.1% [18]. Our study represents the largest population of patients undergoing complex AWR that has been evaluated for VTE. Similar to the studies by Kraft et al*.* and Zhang et al*.*, we retrospectively reviewed prospectively collected data. However, our results were also supplemented with both data extraction using natural language processing and manual chart review for confirmation.

While component separation has previously been identified as a potential risk factor for VTE after VHR, there is no current consensus in the literature. Andriyashkin et al*.* performed a retrospective analysis of prospectively collected data, in which compression duplex ultrasound was performed in a group of 240 patients undergoing incisional hernia repair [5]. They found a 7.9% incidence of VTE in these patients, and on multivariate analysis, component separation was found to be a statistically significant risk factor. However, when Kim et al*.* reviewed ACS-NSQIP data for patients undergoing VHR, component separation was not found to increase the odds of VTE [6]. While our current analysis does not statistically evaluate the association of component separation with VTE development, it does demonstrate a higher incidence of VTE when compared to studies of VHR in general, which range from 0.5 to 0.92% [1–4]. Given VHR with component separation generally encompasses many of the operative characteristics found to increase the risk of postoperative VTE, such as longer operative times and inpatient surgery [3, 14], consideration should be given as to whether these patients should be evaluated as a distinct cohort from other VHR patients.

Perhaps the most important points of discussion are the management strategies for the prevention of VTE, for which a number of questions remain unresolved. In general, perioperative prophylaxis in patients undergoing VHR is directed by CHEST guidelines, which recommend chemoprophylaxis based on the risk of VTE [22]. These guidelines are not specific to surgical procedures, but rather are considered to be applicable to any patient undergoing general, urological, gynecologic, bariatric, vascular, or plastic and reconstructive surgery. Additionally, they do not provide recommendations regarding timing, dosages or length of treatment. While the benefit of perioperative chemoprophylaxis in hernia patients is widely acknowledged, optimal timing and duration have not been defined, resulting in significant variability in practices [23].

Preoperative administration of subcutaneous heparin for the prevention of VTE in surgical patients has been adopted by the Surgical Care Improvement Project (SCIP) initiative as a quality metric [24]. The SCIP recommendation for the administration of pharmacologic prophylaxis within 24 h prior to surgery is largely based on prior high-volume, meta-analyses of randomized controlled trials which found a significant reduction in VTE with preoperative heparin administration [25, 26]. Interestingly, these analyses were composed of trials which only compared preoperative dosing to either placebo or control, not postoperative administration. Within the orthopedic literature, this question examining the benefit of preoperative chemoprophylaxis found that postoperative initiation was no less effective in the prevention of VTE [27, 28]. More recently, two meta-analyses reviewed patients undergoing abdominal surgery and compared the incidence of VTE between preoperative and postoperative initiation of chemoprophylaxis [23, 29]. Both found that the incidence of VTE was not associated with the timing of chemoprophylaxis, and furthermore, early usage was associated with a statistically significant increase in bleeding risk. In fact, Fagarasanu et al*.* found similar results when they specifically reviewed patients undergoing ventral hernia repair [30]. There is sufficient evidence to warrant additional re-investigations regarding the need for early chemoprophylaxis.

After complex AWR, patients are commonly administered chemoprophylaxis only up until discharge from the hospital, at which time it is assumed patients are ambulating and their risk of VTE has returned to baseline. However, the duration of chemoprophylaxis should be discussed within the context of the timing of VTE events. The median time to a VTE event in this analysis was 7 (IQR 4–14) days after surgery and 27% of events occurred after hospital*.* Specifically, within patients undergoing complex AWR, there are limited data on the timing of VTE. Only one study by Kraft and Janis reported the average time of diagnosis, which was at 11.25 days [16]. There are, however, several studies which include patients undergoing any type of VHR where the average time to VTE diagnosis ranged from 10.8 to 15 days [1–3]. Taking this into account, the median length of stay after VHR with TAR has been found to be less than 7 days in most prior analyses [31–33]. This translates into a high likelihood that VTE risk remains high in these patients even after discharge.

Current literature has demonstrated an improved benefit–to-risk ratio of extended thromboprophylaxis for patients undergoing certain operative procedures, such as orthopedic, bariatric or cancer surgery [30, 34–37]. Standard recommendations for patients undergoing one of these specific operations are for 2–4 weeks of extended chemoprophylaxis. Risk calculators have also been created to identify those at particularly high risk who may benefit from chemoprophylaxis beyond hospital admission [38]. When Kumar et al*.* reviewed this question in VHR patients, using ACS-NSQIP data, they identified a subset of patients who were at high risk of VTE after discharge [2]. They found that at a threshold risk of 0.3%, over half of the patients would develop post-discharge VTE and recommended that these patients should be considered for extended chemoprophylaxis. Accordingly, our analysis found that almost one-third of the VTE events occurred after hospital discharge from index admission. While these findings may support the use of extended chemoprophylaxis, this must be balanced with the associated risks of anticoagulation in a patient population that has also previously been shown to have a high risk of bleeding [39]. Future studies should focus on clarifying risk factors and identifying which patients, if any, should be considered for extended chemoprophylaxis after VHR.

This study is not without limitations. First, because this was a descriptive study without hypothesis testing, we cannot make conclusions regarding the association of VTE development with the various perioperative variables that have been described. Additionally, as is the nature of retrospective analyses and manual data extraction of medical records, the data collected are subject to inaccuracies, bias, or missing variables. For example, while our study included both unilateral and bilateral TAR, these data were not available to report. Third, our study had broad inclusion criteria, with the goal of describing a diverse cohort of patients undergoing VHR with TAR. However, this prohibits a deeper analysis of certain high-risk patient populations, and our findings may not be applicable to less complex patients without significant comorbidities. And finally, all data were collected from a single, high-volume, specialized abdominal core health center, which may limit the generalizability of our findings.

## Conclusions

Patients undergoing ventral hernia repair with transversus abdominis release are at high risk for venous thromboembolic events, even after hospital discharge. Additionally, further studies are needed to determine risk factors associated with VTE in this specific patient population and the relative risk of VTE compared to patients undergoing other types of ventral hernia repair. This information can be used to generate guidelines for the optimization of perioperative anticoagulation strategies for complex abdominal wall reconstruction.
